# Ammonia Iron Alum

**Published:** 1868-12

**Authors:** 


### ARTICLE IX.

### "*Ammonia Iron Alum.*"

Ferri and ammonia sulphat, the elegant styptic remedy which has recently been much prescribed in diseases of the mucous membranes, attended with excessive discharge, and for the preparation of which I would refer to works on pharmacy, was first brought to the notice of the Pharmaceutical Society of London, in December, 1853, by Mr. Lindsey Blyth, as a new remedy prescribed in St. Mary's Hospital. Dr. Tyler Smith found it more astringent than the common alum, and devoid of the stimulating effects of iron, and while it controls excessive discharges, is often useful in correcting their cause.

Though called alum this salt contains no alumina, which is replaced by sesqui oxide of iron.

It has been used internally in leucorrhœa, and with benefit in diarrhœa and chronic dysentery, and other affections requiring a continuation of tonics and astringents; when given internally, the dose is from three to twelve grains twice or three times a day.

But on the present occasion I would beg leave especially to direct the attention of the members of this Association to this "ammonia iron alum" as a medicine for controling active as well as passive hæmorrhage. My experience with it in such affections having yielded the most satisfactory results, a few instances may serve to show that the prompt results obtained by this remedy may be a sufficient apology for obtruding this paper upon you.

I was called, September 23, 1861, at sunrise, to Miss S. M. a young lady aged 19, of sanguineo—nervous temperament; had generally been in fine health, but about a week previous had had an attack of bilious intermittent fever for which she had been treated by another physician, and from which she had not entirely recovered, when at ten o'clock the night previous to my visit, she was taken with epistaxis ; the hæmorrhage was profuse and without interruption, in spite of all means to suppress it until I saw her. Her physician being absent, I was sent for, and found her perfectly prostrated with cold extremities, her pulse 130 per minute, and very feeble, her countenance, usually very florid, was deathly pale, and the bleeding still continued from the right nostril. I speedily prepared a saturated solution of the ammonia iron alum, in which I soaked cotton, and with which I plugged the nostril, when the hæmorrhage instantly ceased, nor was there any return of it for two days—the patient rallying promptly under appropriate remedies—when a profuse hæmorrhage from the bowels speedily destroyed life. I have repeatedly applied the remedy in epistaxis and never failed to almost instantaneously stop the discharge. In a case of hæmorrhage from the bowels which occurred last year, in typhoid fever, I checked the discharge promptly with it. But more recently, in a case of cancer of the uterus when an artery was destroyed by ulceration and the hæmorrhage amounted in a few minutes to at least two pints, being accidentally at hand, a tampon of cotton being thoroughly wet with a solution of am. iron alum, at once arrested the hæmorrhage and although the unfortunate patient, who was absent from her family at the time , died by a repetition of the hæmorrhage had at least a delay sufficient, to have the gratification of seeing her friends around her, and preparing for the awful change.

These and many other results have induced me to direct your attention to this remedy, believing it to be a styptic of great value; and knowing that it is but little used by the members of this Asseciation, I respectfully recommend it to their favorable consideration when occasion may offer.—*Cases reported by Drs. Kemp and Leighton. New Orleans Journal of Medicine.*

### MONTHLY SUMMARY.

